# Prevalence and determinants of death registration and certification uptake in Uganda

**DOI:** 10.1371/journal.pone.0264742

**Published:** 2022-03-04

**Authors:** Leonard K. Atuhaire, Elizabeth Nansubuga, Olivia Nankinga, Helen Namirembe Nviiri, Benard Odur

**Affiliations:** 1 Department of Planning and Applied Statistics, School of Statistics and Planning, Makerere University, Kampala, Uganda; 2 Department of Population Studies, School of Statistics and Planning, Makerere University, Kampala, Uganda; 3 Directorate of Population and Social Statistics, Uganda Bureau of Statistics, Kampala, Uganda; 4 Department of Statistical Methods and Actuarial Science, School of Statistics and Planning, Makerere University, Kampala, Uganda; Health Directorate, LUXEMBOURG

## Abstract

Death registration in Uganda remains extremely low, yet mortality statistics are vital in health policy, planning, resource allocation and decision-making. According to NIRA, only 1% of deaths are registered annually, while Uganda Bureau of Statistics estimates death registration at 24% for the period 2011–2016. The wide variation between the administrative and survey statistics can be attributed to the restriction to only certified death registration by NIRA while survey statistics relate to all forms of death notification and registration at the different sub-national levels. Registration of deaths is of critical importance to individuals and a country’s government. Legally, it grants administrative rights in management of a deceased’s estate, and access to social (insurance and pension) benefits of a deceased person. It is also essential for official statistics and planning purposes. There is an urgent need for continuous and real-time collection of mortality data or statistics in Uganda. These statistics are of significance in public health for identifying the magnitude and distribution of major disease problems, and are essential for the design, implementation, monitoring, and assessment of health programmes and policies. Lack of such continuous and timely data has negative consequences for the achievement of both national and Sustainable Development Goals 3, 11, 16, and 17. This study assessed the determinants of death registration and certification, using a survey of 2018–2019 deaths in 2,100 households across four administrative regions of Uganda and Kampala district. Multivariate–binary logistic regression was used to model factors associated with the likelihood of a death being registered or certified. We find that around one-third of deaths were registered while death certificates were obtained for less than 5% of the total deaths. Death registration and certification varied notably within Uganda. Uptake of death registration and certification was associated with knowledge on death registration, region, access to mass media, age of the deceased, place of death, occupation of the deceased, relationship to household head and request for death certificate. There is need for decentralization of death registration services; massive sensitization of communities and creating demand for death registration.

## Introduction

Civil registration is the continuous permanent, compulsory recording of the occurrence and characteristics of vital events and as provided through decree of regulation in accordance with the legal requirement in each country [1]. According to [2] 32% of the countries and territories have less than the desired 90% death registration coverage and by 2015, the global death registration completeness stood at 38.6% [3]. Notable, most people in Africa and Asia neither register births or deaths, which has huge implications for legal identities and a country’s official statistics [4–7]. Given a relatively high birth registration rate and low death registration rate, there may be large numbers of legal identities that are never been removed from the system, such as from electoral rolls, social and health benefits. This carries risks of identity fraud which can be costly to governments and to the economy. Also, the complexity of the death registration processes means that only those interested in certain benefits pursue death certification.

Death registration in Uganda remains extremely low [8], despite the urgent need for continuous and real-time collection of mortality statistics; in addition to its legal benefits to both individuals and Government. At individual level, it is vital in administration of a deceased’s estate, insurance and pension claims while at government level, it is essential for official records or statistics and planning for electoral processes, and access to social benefits. Mortality statistics are vital in health policy, planning, resource allocation and decision-making [9]. In Uganda, complete death registration is essentially a three step process: (i) death notification to the health facility or local administrative authority, (ii) official, legal registration of the death with the civil registration authority, and (iii) certification of the death with issuance of a death certificate. According to National Identification and Registration Authority (NIRA), only 1% of deaths (3,886 deaths) were registered and only 2,893 death certificates were issued between July 2018 and June 2019 [10]. On the other hand, Demographic and Health Survey results collected in 2016 estimated death registration at 24% [8]. The wide variation between the administrative and survey statistics can be attributed to the restriction to only certified death registration by NIRA while survey statistics relate to all forms of death notification and registration at the different sub-national levels. Additionally, demographic survey data refers to reporting of deaths about the 12 month period prior to the survey data collection.

Existing literature categorizes the factors for low uptake of death registration into two–micro and macro related factors. Although micro—related factors like low public awareness are often cited as the key contributory factors for low death registration [11–15], the problem appears to be largely on the macro—side (structural factors). Structural factors include: outdated legal frameworks, overlapping responsibilities, and inaccessible registration services in rural areas, poor collaborative mechanisms amongst stakeholders, poorly designed systems, inadequate training, poor quality control (both coverage and quality of cause of death reporting), lack of political will, complex registration systems, inadequate funding, weak enforcement of laws, ambiguity of roles, limited use of technology, poor attitude of registration officials, and non-integrated systems [13, 15–20].

We used the theory of planned behavior [21] to examine the determinants of death registration in Uganda. In this study, the theory of planned behavior explains the motivation and intentions of individuals to partake in death registration and certification. According to this theoretical framework, understanding the intentions for death registration and certification is influenced by individual attitudes, subjective norms (perceived social pressure), and perceived behavioral control. Perceived behavioural control may directly or indirectly influence behavior either through one’s intentions or perceived barriers and facilitators of death registration and certification. At individual level, employment status of the household head, residence, age at death, household wealth [14, 17, 22], educational status of the household head [12], refugee status, and age at death [17] are identified as key determinants of death registration.

Lack of a vibrant civil registration mortality system in Uganda negatively impacts the achievement of health related national and sustainable development goals. Sustainable Development Goal (SDG) 3 (targets 3.1–3.4, 3.6, 3.9), SDG 11 (target 11.5) and SDG 16 (target 16.5) advocate for ending or reduction of deaths due to various causes while SDG 17 (17.8, 17.9) advocates for timely, reliable data, monitoring and accountability. All these targets require continuous compilation of mortality data which is often incomplete or lacking. An efficient civil registration provides the most reliable source of health data for measuring and monitoring these SDG indicators [3, 23]. Additionally, Uganda’s health sectoral development plan prioritizes a more comprehensive health information system including death registration as a strategic focus area that is critical for the achievement of universal health coverage [24]. This national health plan also highlights the critical gap and need for comprehensive strategies towards data generation and validation using systems such as civil registration and vital statistics. Thus, this research fills a critical knowledge gap and generates data on death registration. Therefore, the main objective of this study was to estimate the prevalence and examine the determinants of death registration uptake in Uganda.

## Data and methods

This study is part of a larger mixed methods (quantitative and qualitative) study employing a cross–sectional research design. This particular research study is based on primary data obtained through quantitative data collection techniques. A sample was collected from each of the four administrative regions of Uganda and the capital city Kampala. In each region, one district was randomly selected. Bushenyi, Mayuge, Lira and Mityana districts were selected from the Western, Eastern, Northern and Central regions respectively. The regions are culturally distinct. The Central region is majorly comprised of Baganda ethnic grouping, Western region—Banyankore, Eastern region–Basoga ethnicity while Northern region is comprised of mainly Acholi and Lango ethnic populations. Additionally, data was collected from Kampala district (found in Central region) due to its uniqueness as the capital city, high level of urbanization and diverse population. Each ethnic grouping has cultural beliefs, practices, norms regarding death, mourning and bereavement which may have implications on death registration and certification.

The inclusion criteria for the study population was occurrence of a death in a household in in 2018 and 2019. Non–probability quota sampling was used to achieve the required sample size. In each district, four (4) sub-counties were selected at random, taking into account the rural urban residence. Each selected sub-county was traversed identifying and recruiting eligible households until the required quota was achieved in the district. From each sampled household, one respondent preferably a household head aged above 18 years was interviewed. The household head was the preferred respondent because he/ she would have all the information pertaining to the household. However, in the absence of a household head, the spouse or any adult aged18 years was eligible to respond to the interview questions.

A sample size of 420 households was targeted from each district, thus a total of 2100 respondents from five districts. This sample size was obtained by using Cochran’s formula, and assuming a death registration prevalence of 25%, required precision of ± 0.05, a 95% confidence level, and a design effect of 1.5. A sample size of 2,044 respondents was achieved, which was considered to be a nationally representative sample.

To achieve the desired sample size, computer–assisted personal interviewing technique using open data kit software was employed for data collection. A team of 20 trained research assistants, 5 field supervisors, and 3 technical supervisors conducted the study.

Data was collected on the demographic, socio-economic characteristics of the household head (age, sex, religious affiliation, marital status, number of household members, household assets, educational attainment, occupation), access to sources of information/ communication (phone, television, radio, internet), and ownership of transport means. In addition, data was collected on the deceased’s characteristics (age, sex, occupation, relationship to household head, cause of death as reported by the respondents) on information on death registration (knowledge, importance, reasons for registration or non-registration, type of document acquired for death notification or registration), and other potential determinants of death registration as shown in Table 1. The outcome variable was death registration, whether a death was registered or not.

**Table 1:** Description of death registration variables.

Variable	Description	Data type	Data coding
Death Registration	Whether occurrence of death in household had been registered or not. *In this study*, *death registration referred to all forms of death notification and registration while death certification referred to issuance of death certificates*.	Nominal	0 = No
			1 = Yes
Knowledge of Death Registration	This related to respondents’ awareness of the meaning, importance and benefits of death registration. Respondents who knew all the three aspects of knowledge were classified as having comprehensive knowledge and likewise.	Categorical	1 = None
			2 = Partial
			3 = Comprehensive
Documents regarding death	Whether a respondent had any documentation regarding the deceased death.	Nominal	1 = Yes
			2 = No
Type of Document	For respondents who had any form of documentation, these related to the type of documentation that one had.	Nominal	1 = Death Certificate
			2 = Postmortem Report
			3 = Health facility report
			4 = Sub county report
			5 = Local council letter
			6 = Others
Ever been asked for death certificate	Whether respondent had ever been asked to present a death certificate to access any service	Nominal	0 = No
			1 = Yes
Aware of Penalty	Whether respondent was aware of any penalties for non death registration	Nominal	0 = No
			1 = Yes
Cause of death	This was respondent reported cause of death of the deceased persons, based on recall. Classification was given for the common causes of death. In some cases, the respondents were unaware of the cause of death. Cause of death was in most cases not medically certified, and only verified where medical records were available.	Nominal	1 = Pregnancy related
			2 = Malaria
			3 = Mental illness
			4 = HIV/AIDS
			5 = Cancers
			6 = Heart diseases
			7 = Respiratory diseases
			8 = Accident
			9 = Congenital illness
			10 = Witchcraft
			11 = Others (Specify)
			12 = I don’t know
			13 = Diabetes
			14 = Anaemia
			15 = Liver illness
			16 = Kidney illness
			17 = Childhood illness
			18 = Digestive system illness
Place of death	This referred to the place where the deceased died from. This was categorized into four categories. Others referred to persons who died on the way, accidents, by the roadside and any other unlisted place.	Nominal	1 = Home
			2 = Private health facility
			3 = Government health facility
			4 = Others
Relationship to the deceased	Respondents’ relationship to the deceased person	Nominal	1 = Spouse
			2 = Child
			3 = Sibling
			4 = Parent
			5 = Parent in law
			6 = Other
Age	Data on age was recorded in single years and was later recoded/ regrouped during data analysis.	Count	>18 years
	Data was collected on age of respondent and also age of deceased person at the time of death.		

Data analysis was done using STATA (version 15) software. Prevalence of death registration and certification was estimated by computing the proportion of deaths out of the total deaths that occurred in 2018 and 2019 that were registered and certificates issued, respectively. Analysis was done at three levels: univariate, bivariate and multivariate levels. Descriptive statistics were presented at univariate level. The relationship between death registration uptake and the different variables was initially assessed using the Pearson chi-square test (χ2) at bivariate level while at multivariate level, binary logistic regression was used to model the likelihood of a death being registered/certified. The level of significance was set at 0.05 and 95% confidence intervals were used. Only variables that were significant at bivariate level (p < 0.05) were considered in the multivariate level analysis. The final model included only variables that were significant. Regression diagnostics were run to assess the goodness of fit before selecting the final model.

### Ethical considerations

Ethical approval was granted by The AIDS Support Organization Institutional Review Board and Uganda National Council for Science and Technology. Permission to undertake the study in the selected districts was also sought from the various district local governments/ administrative authorities at the various levels. Furthermore, informed consent was also sought from the respondents who were made aware of the study purpose, procedures, benefits, as well as the intended use of the data. Each questionnaire was accompanied by a signed consent form. Data analysis has been done at aggregate level.

## Results

### Description of respondents and death registration and certification

Table 2 presents the descriptive and bivariate results. Almost an equal proportion of respondents (21%) were interviewed in each of the study districts. The average size of a household was 6.1 persons and the average age of the household head was 50.2 years. The highest proportion of respondents had primary education (54%), were Catholics (38.1%), married or cohabiting (52.1%), and were peasant farmers (55.4%). Only 40% of respondents had access to means of transport (car, truck, van, motorcycle, scooter or bicycle) while 90% of the households owned at least one form or means of communication (radio, computer, mobile phone, internet, newspapers or television).

**Table 2:** Distribution of respondents and uptake of death registration and certification.

Variables	Percent of frequency	Frequency	Percent of deaths registered (DR)	Percent of deaths with certificates (DC)
**District**		**p-value 0.000**		**p-value 0.000**
Kampala	20.7	422	54.5	14.7
Mityana	21.0	429	40.3	2.1
Bushenyi	17.0	347	21.6	1.4
Lira	20.6	420	25.7	1.9
Iganga	20.9	426	22.1	1.6
**Education level**		**p-value 0.000**		**p-value 0.000**
None	16.1	329	21.6	0.9
Primary	54.0	1103	30.6	2.2
Secondary	23.3	476	41.0	8.0
Tertiary	6.7	136	55.9	19.1
**Religion**		p-value 0.053		**p-value 0.012**
Catholics	38.1	778	32.4	4.1
Anglican	32.1	657	31.1	2.6
Pentecostals	10.6	216	40.7	7.4
Muslims	17.4	355	33.2	6.5
Seventh Day Adventists	1.3	27	44.4	7.4
Other	0.5	11	54.6	9.1
**Occupation of Head**		**p-value 0.000**		**p-value 0.000**
Peasant	55.4	1132	26.9	12.5
Housewife	2.0	40	52.5	1.9
Own Business	27.9	571	40.8	6.3
Private Employment	7.4	152	37.5	6.6
Government Employment	2.2	45	46.7	17.8
Unemployed	5.1	104	41.4	9.6
**Own Transport means**		p-value 0.413		p-value 0.404
Yes	39.8	813	34.3	4.9
No	60.2	1231	32.6	4.1
**Access to Communication means**		**p-value 0.000**		**p-value 0.001**
Yes	89.6	1832	34.9	0.0
No	10.4	212	18.9	5.0
**Marital Status**		p-value 0.093		**p-value 0.000**
Never Married	4.9	101	43.6	10.9
Married/ Cohabiting	52.1	1064	31.7	2.8
Divorced/ Separated	8.2	168	32.7	1.8
Widowed	34.8	711	34.3	6.6
**Total**	**100**	**2,044**	**33.3**	**4.5**
**Aware of any Penalty -**		**p-value 0.000**		**p-value 0.000**
No	96.3	1969	32.4	3.9
Yes	3.6	74	56.8	20.3
**Ever been asked for a death certificate**		**p-value 0.000**		**p-value 0.000**
No	92.3	1886	29.7	1.0
Yes	7.7	157	76.4	46.5
**Knowledge Levels**			**p-value 0.000**	**p-value 0.000**
None	57.2	1,170	27.3	0.9
Partial	28.6	584	36.5	5.1
Comprehensive	14.2	290	51.0	17.6
**Total**	**100**	**2,044**	**33.3**	**4.5**
**Documents regarding Death**				
Yes	40.1	819		
No	59.9	1224		
**Type of Document**				
Death Certificate	11.1	91		
Postmortem report	4.5	37		
Health Centre Death report	65.3	535		
Sub county report	2.1	129		
Local council letter	15.8	10		
Other	1.2	17		
**Reasons for Registration**				
Access to Pension	22.4	17		
Inheritance	32.9	25		
Access Insurance	10.5	8		
Legal Requirement	15.8	12		
Other	18.4	14		
**Reasons for Non Registration**				
Too much costs	3.0	58		
Long distance travel	1.1	21		
Did not know it’s a requirement	70.8	1393		
Did not know where	9.2	181		
Did not know why	6.2	122		
Other reason	9.8	192		
**Ever registered a death**				
No	97.2	1986		
Yes	2.8	57		
**Total**	**100**	**2,044**		

Average Age of Household Head 50.2 years (Standard Deviation 16.3)

Average Household size 6.1 members.

Only 3.6% of the respondents were aware of any penalty associated with non-registration of deaths. Additionally, 7.7% of the respondents had ever been asked to present a death certificate while only 2.8% have ever registered a death in their lifetime. Only 40% of the respondents had a document regarding the deceased’s death, which mainly included health facility reports (65.3%). The main reasons for death registration were inheritance and access to deceased’s pension. Majority of the respondents (70.8%) were ignorant about the registration of deceased persons.

Table 2 also presents results on the relationship between the respondents’ characteristics and death registration uptake. Death registration and certification are significantly associated with district, educational attainment, occupation, access to communication means, awareness of death registration penalty, knowledge and ever been asked for a death certificate (p< 0.05). Similarly, there is a significant relationship between death certification and religion, marital status (p< 0.05). There is significant regional variation in uptake of death registration. Death registration is highest in Kampala district (54.5%), and among those with tertiary education (55.9%) and comprehensive knowledge. Deaths reported by housewives had the highest registration proportion (52.5%) followed by those reported by government employees (46.7%). Death registration was also highest by respondents who had access to any means of communication (34.9%), were aware of penalties for non—death registration (56.8%), and respondents who had ever been asked for a death certificate (76.4%). Death certification is highest in Kampala district (14.7%), among respondents with tertiary education (19.1%), non Christian and non Muslim religions (9.1%) and those with comprehensive knowledge on death registration (17.9%). Additionally, death certification is highest among respondents who are government employees (17.8%) and peasants (12.5%), never married (10.9%), those aware of penalties for non—death registration (20.3%), and those who have ever been asked for a death certificate (46.5%). This could be attributed to knowledge on death registration by government employees, and need to access the deceased’s estate including customary or family land by peasants.

Table 3 presents the descriptive and bivariate results of the deceased persons and uptake of death registration and certification. The largest categories of deceased persons were males (58.5%), those aged 70 years and above (25.5%), peasant farmers (36.8%), those who died from home (46.5%) and those whose main causes of death were heart diseases (11.7%) and malaria (11.3%). In terms of relationship to the head of household, the largest category of the deceased were children to the household heads (32.9%) followed by 23.1% who were spouses.

**Table 3:** Percent distribution of characteristics of deceased persons by death registration & certification.

Characteristics	Percent (%)	Frequency	Percent of deaths registered (DR)	Percent of deaths with certificates (DC)
**Cause of Death**		**p-value 0.000**		**p-value 0.000**
Pregnancy related complications	2.3	48	50.9	6.3
Malaria	11.3	230	30.0	0.9
Mental Illness	0.7	15	33.3	6.7
HIV/AIDS	8.6	176	30.1	1.7
Cancers	8.7	177	37.8	8.5
Heart Diseases	11.7	240	33.7	6.3
Respiratory diseases	10.5	214	34.6	3.7
Accident	7.2	147	41.5	8.8
Congenital Illness	4.7	96	30.2	0.0
Witchcraft	3.5	71	19.7	1.4
Diabetes	3.6	73	43.8	8.2
Anaemia	1.7	34	38.2	2.9
Liver disease	1.7	34	52.9	5.9
Kidney disease	1.2	26	50.0	19.2
Childhood illness	1.5	31	25.8	3.2
Digestive system disease	2.6	53	26.4	0.0
Others	9.2	189	33.0	6.4
I don’t Know	9.3	190	21.6	1.6
**Place of Death**		**p-value 0.000**		**p-value 0.000**
Home	46.5	950	10.8	2.1
Private Health Facility	13.8	282	51.8	7.8
Government Health Facility	33.8	691	57.5	5.5
Other	5.9	121	28.1	9.1
**Age of the Deceased**		**p-value 0.003**		**p-value 0.000**
<1	7.3	149	36.2	0.0
1–4	7.7	158	27.9	1.3
5–9	3.6	73	31.5	0.0
10–19	4.6	94	30.9	2.1
20–29	9.6	196	41.8	4.6
30–39	11.5	235	31.5	4.3
40–49	11.6	237	33.3	1.7
50–59	9.8	201	42.8	12.9
60–69	8.8	180	35.6	10.0
70+	25.5	521	27.8	3.8
**Sex of the deceased**			p-value 0.642	**p-value 0.011**
Male	58.5	1196	32.9	5.4
Female	41.5	848	33.8	3.1
**Relationship to the head**			**p-value 0.008**	**p-value 0.000**
Spouse	23.1	472	38.9	9.8
Child	32.9	672	34.4	1.9
Sibling	10.6	217	28.6	3.7
Parent	18.3	374	32.1	5.6
Parent in law	3.8	78	25.6	2.6
Other	11.3	231	27.3	0.4
**Occupation of Deceased**			**p-value 0.000**	**p-value 0.000**
Housewife	2.1	42	54.8	14.3
Peasant	36.8	753	24.4	1.2
Own Business	19.6	400	41.3	7.3
Private Employee	5.8	119	42.0	8.4
Government Employee	3.8	77	55.8	28.6
Unemployed	9.2	188	36.7	5.3
Not Applicable (<15 years)	22.7	4645	-	1.1
Not Known	0.05	1	31.5	0.0
**Total**	**100**	**2,044**	**33.3**	**4.5**

Death registration and certification are significantly associated with cause of death, place of death, relationship to household head, age of deceased and occupation of the deceased (p< 0.05). Additionally, death certification is significantly associated with age and sex of the deceased (p < 0.05). Deceased government employees and spouses to household heads recorded the highest proportion of death registration and certification. Although, a third of male and female deaths (33%) were registered, death registration was generally highest among persons who died from government health facilities (57.5%). Deaths from causes which are very precisely defined such as liver (52.9%), kidney (50%) and pregnancy (50.9%) complications reported a higher proportion of registered deaths. Notably, there is a low death registration rate for those believed to have died of witchcraft (1.4%)! This finding reflects the fact that persons who die from a health facility where a doctor completes the WHO Medical Certificate of Cause of death" are more likely to be notified to the civil registrar and then officially registered. On the other hand, deaths believed to be due to witchcraft are likely to have occurred at home and are less likely to be registered than those occurring in a health facility. Additionally, death certification was highest among deceased males (5.4%), persons who died of kidney complications (19.2%) and those who died from accidents or outside home or health facilities (9.1%). The proportion of registered deaths was highest for deceased persons who were aged 50–59 years (42.8%) followed by those aged 20–29 years (41.8%). Death certification was highest for older deceased persons aged 50–59 years (12.9%) and 60–69 years (10%).

### Prevalence of death registration and certification

**Fig 1:**
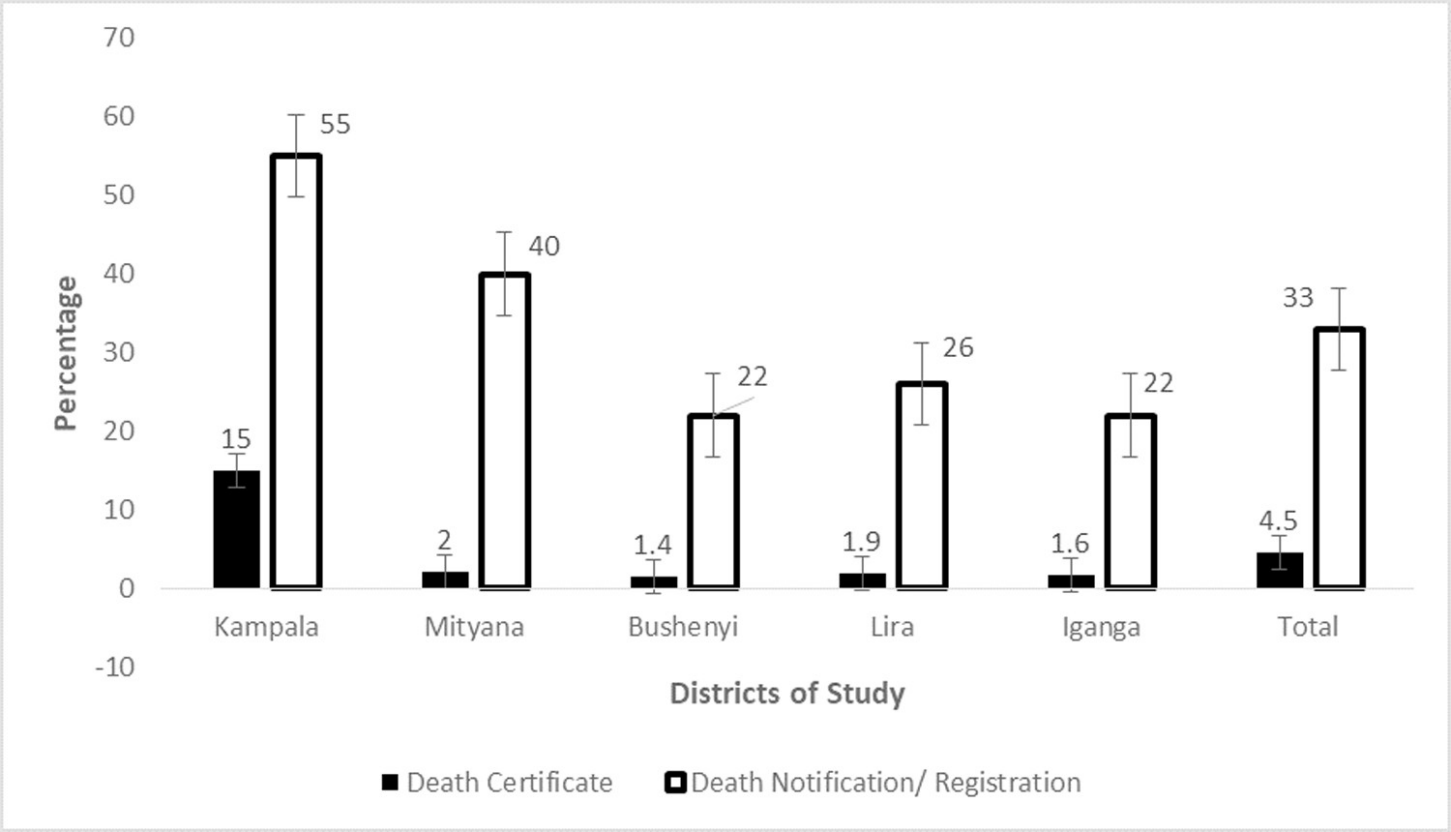
Prevalence of death certification and registration.

Death registration and certification remains extremely low in Uganda, despite its critical importance. A third of the deaths (33%) that occur in Uganda are registered or notified at different levels. However, only 4.5% of deaths that occur in Uganda are legally registered by obtaining a death certificate as shown in Fig 1. Death registration and certification is highest in Kampala district followed by Mityana district as shown in Fig 1. In all other districts, about 2% of deaths were registered and a death certificate obtained. The low uptake of death registration and certification can largely be attributed to ignorance or lack of knowledge on death registration, its importance and ignorance on places where death registration is done.

### Association between death registration and certification uptake and explanatory factors

Table 4 presents the results of the binary logistic regression model that was used to examine the relationship between explanatory variables and the likelihood of death registration and certification. Respondents in Bushenyi, Lira and Iganga had reduced odds of registering a death compared to respondents in Kampala (AOR = 0.19; CI 0.13–0.28; AOR = 0.25; CI 0.17–0.37 and AOR = 0.23; CI 0.15–0.33 respectively). Higher odds of registering a death were observed for respondents who had access to media than those who did not (AOR = 1.14; CI 1.05–1.23). Results showed that respondents with average (AOR 1.37, 95% CI 1.05–1.80) and comprehensive (AOR 1.68, 95% CI 1.17–2.40) knowledge on death registration had higher odds of registering a death than those without any knowledge. Respondent who reported that they were ever asked for a death certificate had higher odds of registering a death compared to those have never been asked for the certificate (AOR 4.64, 95% CI 2.90–7.40). Place of death was also significantly associated with registration of a death. Notably, deaths that occurred at government and private health facilities were 16.4 times and 12.8 times more likely to be registered as compared to those that occurred at home.

**Table 4:** Determinants of death registration and death certification.

Determinants	Death Registration (Odds Ratio)	Confidence Interval (C.I)	Death Certification (Odds Ratio)	Confidence Interval (C.I)
**District**				
Kampala (Ref)	1.000		1.000	
Mityana	0.731	0.514–1.039	0.177***	0.061–0.515
Bushenyi	0.188***	0.126–0.280	0.188***	0.048–0.742
Lira	0.253***	0.171–0.374	0.133***	0.038–0.469
Iganga	0.227***	0.154–0.334	0.121***	0.034–0.429
***Access to Media***				
No (Ref)	1.000			
Yes	1.136***	1.048–1.230		
**Comprehensive Knowledge**				
No Knowledge (Ref)	1.000		1.000	
Partial/ Average Knowledge	1.373***	1.049–1.796	1.790	0.704–4.547
Comprehensive Knowledge	1.676***	1.169–2.405	7.967***	3.021–21.011
**Place of Death**				
Home (Ref)	1.000		1.000	
Private health facility	12.824***	8.859–18.563	2.565	0.947–6.950
Government health facility	16.354***	12.104–22.096	1.844	0.762–4.461
Others	3.051***	1.828–5.091	4.471***	1.293–15.458
**Ever asked for death certificate**				
No (Ref)	1.000		1.000	
Yes	4.640***	2.908–7.403	47.310***	22.151–101.043
**Age of Deceased**				
0 (Ref)	1.000		1.000	
1–4	0.940	0.534–1.655	2.738	0.136–55.086
5–9	1.021	0.505–2.063	1	
10–19	1.103	0.571–2.134	3.497	0.242–50.587
20–29	1.555	0.917–2.636	0.776	0.155–3.884
30–39	1.133	0.674–1.903	1.292	0.312–5.353
40–49	1.212	0.723–2.030	0.173***	0.034–0.880
50–59	1.578	0.930–2.679	2.123	0.678–6.646
60–69	1.661	0.955–2.889	3.782***	1.21–11.787
70+	1.520	0.951–0.429	1	
**Relationship to Head**				
Spouse (Ref)			1.000	
Child			0.435	0.121–1.560
Sibling			0.304	0.086–1.082
Parent			0.487	0.189–1.254
Parent in law			0.681	0.107–4.319
Other			0.36***	0.003–0.406
**Occupation of Deceased**				
Housewife (Ref)			1.000	
Peasant			0.089***	0.018–0.448
Own Business			0.138***	0.033–0.570
Private Employee			0.108***	0.200–0.581
Government Employee			0.644	0.139–2.989
Unemployed			0.138***	0.025–0.761
Not Applicable (<15 years)			0.093	0.007–1.263
**Cons**	0.067	0.033–0.134	0.004	0.155–0.460

*** <0.005.

Death certification was significantly associated with district, knowledge, place of death, ever been asked for a death certificate, age of deceased, occupation of the deceased, and relationship to the household head as shown in Table 4. Respondents in Mityana, Bushenyi, Lira and Iganga had reduced odds of death certification compared to respondents in Kampala (AOR = 0.18; CI 0.06–0.52; AOR = 0.19; CI 0.05–0.74; AOR = 0.13; CI 0.04–0.47 and AOR = 0.12; CI 0.03–0.43 respectively). Death certification was also significantly higher among respondents having comprehensive knowledge (AOR 7.9, 95% CI 3.02–21.01) compared to those who did not. Death certification was also higher for persons who died away from home or health facilities such those who died on the way to a health facility or through road traffic accidents (AOR 4.5, 95% CI 1.29–15.46). Respondents who had ever been asked for a death certificate had higher odds of death certification (AOR 47.3, 95% CI 22.15–101.04) compared to those have never been asked for the certificate. While, the likelihood of death certification for deceased persons aged 40–49 years was lower (AOR 0.17, 95% CI 0.03–0.88), it was higher for deceased persons aged 60–69 years (AOR 3.8, 95% CI 1.21–11.79) compared to infants (less than one year). Occupation was significantly associated with death certification whereby deceased persons who were either peasants, unemployed, private employees or those who owned their business were less likely to have their deaths certified as compared to those who were housewives. Lastly, death certification was lower among deceased persons who had distant relationships with the household head (AOR 0.36 (95% CI 0.003–0.406).

## Discussion

Uptake of death registration and certification in Uganda is low and this has implications on the achievement of national and global mortality goals and targets. Our study findings are in agreement with [8]. The large discrepancy between the proportion of deaths registered and death certification can partly be attributed to ignorance about the death certification processes, high level of bureaucracy in acquisition of a death certificate, high costs incurred and also acceptance of various forms of death registration for claiming benefits. The low uptake can be attributed majorly, to the micro level factors that explain one’s motivation or intention to register a death and acquire a death certificate. Similarly, in line with the theory of planned behaviour, it can be postulated that intentions determined by individual attributes, attitudes, social norms in the various districts, and perceived behavioural control based on one’s knowledge, access to mass media, and place of death influence death registration and certification in Uganda. The study findings have both programme and policy implications on the death registration and certification environment in Uganda.

The higher rate of death registration and certification in Kampala does not only highlight the socio-cultural context but also the wide divide and inequality in access to civil registration services across districts, yet mortality is higher in these under–served districts. The high rate of death registration in Kampala is five–fold. First, ease of accessibility of NIRA offices where death registration services are offered as compared to other districts without functional civil registration services. Notably, Kampala district has several registration offices which are also more accessible unlike in other districts. Past studies have found that distance has an association with access to services [25, 26]. Secondly, the procedures of processing a death certificate are more streamlined and functional at the main NIRA offices and sub–division offices in Kampala as compared to other districts. Such streamlining of processes reduces the extra costs such as those incurred at sub-county level. Thirdly, persons residing in Kampala are more likely to belong to households of a higher socio-economic status [8] and hence have resources to pay for the associated indirect costs (transport) in death registration and certification. Additionally, most deaths in Kampala are more likely to occur at a health facility and thus cause of death would be certified and ease of procession of death registration or certification. Lastly, knowledge about the importance of death registration is higher among Kampala residents coupled with higher educational attainment levels, hence the high uptake of death registration in Kampala district. If the wide disparity in access to death registration services is not urgently addressed, the mortality data derived from vital statistics would continue to present a biased or untrue picture of the mortality situation across the country. The low uptake of death registration and certification in other districts can also be attributed to socio-cultural reasons, where it is a taboo in most cultures to disturb the “spirit or peace of the dead.” Similarly, a study in Kenya reported that newborn deaths are less likely to be registered because culturally they are seen as a bad omen [27]. Access to media increases the likelihood of uptake of death registration in Uganda. Mass media increases the transmission of knowledge and health related information [28, 29]. Thus, mass media is a key strategy to utilize in sensitizing the masses about death registration. This is confirmed by the results that knowledge on death registration including place to register a death, importance of death registration is significantly associated with uptake of death registration.

Place of death is significantly associated with uptake of death registration. Deaths that occur at home are less likely to be registered [12]. The high uptake of death registration for deaths that occur at government health facilities can possibly be due to close collaboration between NIRA and Ministry of Health. These health facilities issue death notification forms (on behalf of NIRA) which aids completion of the death registration processes [10]. Also, the occurrence of these deaths can easily be verified, and cause of death certified which information is critical for completion of death registration. The low registration and certification of deaths that occur at home may be attributed to lack of specific registration and certification guidelines that have to be followed in case of home deaths. It is also plausible that households that take patients to hospitals belong to a better socioeconomic class and can consequently afford the costs incurred in the death registration and certification processes. Being asked for a death certificate is also significantly associated with uptake of death registration in Uganda. This means being asked for a death certificate to access services such as education scholarship (for orphans), pension, life insurance, or administration of a deceased estate can act as a motivation for individuals or influence one’s intention to register a death and acquire a death certificate, as postulated in the theory of planned behaviour. This finding has potential to influence death registration and certification policy and programming in line with linking death registration to access to services in the country. Our finding is in agreement with another study which found that creating demand for the death certificates in order to access other services has the potential of improving death registration [30].

Age of the deceased is significantly associated with uptake of death certification. Death certification was significantly higher among older persons aged 60–69 years. This can be attributed to the associated benefits such as inheritance [31], social benefits, and insurance. This is the case especially if the parent had a higher income [32] or material wealth [33]. The findings are in agreement with studies in Kenya [34] and Senegal [35] which also found a significant association between age of the deceased and death registration and certification.

The low levels of death certification for distant relations to the household head and deceased persons who were peasants, unemployed, or privately employed can possibly be attributed to lack of financial or material gains and hence no motivation for death certification. Deceased persons who were not closely related to the household head are less likely to have death certificates issued. The household head may lack motivation to register such deaths since he or she may not be a direct beneficiary or next of kin for the deceased persons.

The strength of this study lies in its contribution to policy and programming of death registration in Uganda. Its contribution is majorly two–fold. The research expounds on the scientific literature on the prevalence and determinants of death registration and certification; which area is under researched in sub Saharan Africa. It also provides an opportunity of translating research findings into practice. These findings did provide evidence based data that contributed towards the development of a strategic plan for the civil registration authority in Uganda. The findings can also be applied in revitalizing death registration and certification for the rest of sub Saharan Africa.

### Data limitations

The research findings should be considered in light of some limitations with the study. The study districts did not, coincidentally, include underserved and hard to reach districts (mountainous, island districts, border districts, refugee populations, underserved districts—Karamoja region, minority populations—Batwa) which districts may have unique challenges or experiences with death registration. Additionally, collection of information on cause of death which was not verified but captured as stated by the respondents, hence limited analysis on cause of death. Lastly, the study did not probe the causal or proximate determinants of under–registration directly.

## Conclusions

Death registration and certification are significantly associated with district of residence, place of death, knowledge on death registration and being asked for a death certificate. Additionally, death registration is also associated with access to media while death certification is also associated with the deceased’s age, relationship to the household head and occupation. For improvement of death registration and certification, there is need for massive sensitization of communities about death registration regarding the laws guiding death registration, its importance, purpose, and process for acquiring a death certificate. NIRA should foster private–public partnerships as a means to increase uptake of death registration for deaths that occur in the private health facilities and also in the communities. At community level, NIRA needs to engage Village Health Teams and village local council chairpersons to notify deaths that occur in the communities. There is need to decentralize CRVS at sub-national level thereby bringing services near to the communities. There is urgent need for close collaboration between NIRA, Ministry of Health, and Ministry of Local Government in a bid to increase death registration and certification regardless of place of death occurrence. Furthermore, demand should be created for death registration through burial permits which can be issued by the local council chairpersons. Government should also ensure that access to services such as education scholarship (for orphans), deceased’s pension, life insurance and estate administration should strictly be tagged to death registration and certification. The population needs to be encouraged to process death certificates for all deceased persons regardless of age, relationship to household head and occupation. Lastly, there is need for further research to understand intra district variation of death registration and certification in Uganda.

## Supporting information

S1 File Data. (CSV)

S2 File Variable information. (XLSX)

## Abbreviations

CRVS: Civil Registration and Vital Statistics
MoH: Ministry of Health
NIRA: National Identification and Registration Authority
SDAs: Seventh Day Adventists
UBOS: Uganda Bureau of Statistics
UN: United Nations
UN DESA: United Nations Department of Economic and Social Affairs
UNECA: United Nations Economic Commission for Africa
WHO: World Health Organization
